# Challenging the paradigm of alpha-synuclein’s role in disease: can low levels also be bad for you?

**DOI:** 10.1093/sleep/zsae229

**Published:** 2024-09-30

**Authors:** Stefan Clemens, Tonya N Zeczycki

**Affiliations:** Department of Physiology, Brody School of Medicine, East Carolina University, Greenville, NC, USA; Department of Biochemistry and Molecular Biology, Brody School of Medicine, East Carolina University, Greenville, NC, USA

α-Synuclein (α-Syn) is well-known for its connection with neurodegenerative diseases like Parkinson’s (PD), multiple system atrophy (MSA), and dementia with Lewy Bodies. For these and other synucleinopathies, numerous studies indicate high α-Syn levels correlate with both disease pathology and progression [1, 2]. Importantly, deficiencies in synaptic transmissions observed in response to α-Syn knockdown or overexpression coupled with α-Syn’s localization and association with presynaptic terminals and synaptic vesicles highlights one potential functional role this enigmatic protein has in regulating neurotransmitter release and facilitating normal synaptic function and plasticity [3].

Aberrant accumulation of cytosolic α-Syn is thought to promote—via a positive feedback loop—its toxic oligomerization and aggregation, thereby facilitating α-Syn’s pathological interactions, ultimately driving neurodegenerative disease progression [4]. Collectively, these results indicate too much α-Syn is bad for your health. As such, there has been a push to use increased α-Syn levels and their aggregation as a biological marker for the prediction and diagnosis early on for these devastating diseases. Pathological levels of α-Syn have been successfully determined from neuronal and glial cells, or serum [5].

However, there is also evidence that α-Syn levels can decline as part of the normal aging process [6]. Plasma α-Syn concentrations were found to be higher in younger individuals, with levels markedly falling after the age of 35 years. This aging-associated decline in α-Syn could be attributed to normal aging processes and further suggests that imbalances in α-Syn levels beyond a normal non-pathological range might contribute to neuronal vulnerability in otherwise healthy, older individuals. As α-Syn’s functional roles are thought to include maintaining cognitive health and preventing motor control impairment, could this mean that pathologically low levels of α-Syn may also be problematic?

In fact, a recent study by Tahanis et al. (*SLEEP*, in press [7],) does suggest exactly this. In an attempt to identify if α-Syn levels were altered in restless legs syndrome (RLS), the authors compared α-Syn serum levels and clinical data from 113 RLS patients and 45 age- and sex-matched controls. RLS is a highly prevalent sensorimotor disorder that is characterized in part by an urge to move the legs and is often accompanied by periodic limb movements during sleep [8]. The origins of RLS remain unclear, but genetic factors, gender, age, and altered iron homeostasis appear to be important components in the emergence of the pathophysiology.

Using quantitative enzyme-linked immunosorbent-based assays (ELISAs), Tahanis et al. found that α-Syn serum levels were significantly reduced in RLS patients compared to healthy controls. The measurements also revealed that these changes were independent of the sex (male or female) or the age of symptom onset. While these results apparently conflict with the general idea that neurological disorders are associated with elevated levels of α-Syn, several studies have previously indicated that the relationship between RLS and α-Syn levels may deviate from this paradigm. For example, an α-Syn promotor showed a diminished frequency in RLS [9], and a previous postmortem study of RLS patients reported that α-Syn immunohistochemistry was uniformly negative. This implies that α-Syn pathologies normally associated with synucleopathies were not a component of RLS [10]. Together, these findings suggest that there may be a “normal and physiological” range for low α-Syn levels and that aberrations to either side can lead to the emergence of a pathophysiological phenotype.

In addition to the tier 1 level findings of a significant and nearly 30% reduction in α-Syn levels in RLS patients, Tahanis et al. also found that this decrease correlated well with a reduction in serum ferritin levels. Lower ferritin levels, an indicator of systemic iron deficiency, are expected to be found in RLS patients, and low ferritin levels can often be rescued with iron treatments; in addition, iron infusion or oral iron are some of the recommended treatment options for patients with RLS. In their study, Tahanis et al. exposed a subset of 9 RLS patients to iron therapy and found that, predictably, ferritin levels were restored to values comparable to the healthy controls. In a surprising twist of events; however, the iron treatment also increased serum α-Syn levels to those of the age-matched, healthy controls. These are the first data suggesting that iron levels and its homeostasis may be closely linked to α-Syn levels.

Iron homeostasis is tightly regulated, in part, by iron regulatory proteins (IRPs). These cytosolic RNA-binding proteins bind to iron-responsive elements located in the 5’-untranslated regions of ferritin, thereby inhibiting ferritin synthesis. Conversely, under iron overload conditions, the IRPs bind to intracellular iron, dissociate from the iron-responsive elements, and allow for ferritin protein synthesis [11]. Intriguingly, α-Syn also has iron-responsive elements that follow an iron-mediated expression mechanism similar to ferritin [12]. Thus, the authors reveal two novel important aspects in RLS: (1) they support the notion that α-Syn may have a functional role in the disorder and (2) they show that the restoration of normal levels of α-Syn is associated with an improvement of the pathophysiology.

These compelling findings may have important repercussions beyond the field of RLS: As outlined above, α-Syn is upregulated in PD, MSA, and dementia with Lewy Bodies, which led to the concept that only low levels of α-Syn may be good. However, as the Tahanis et al. paper now points out, very low levels of α-Syn may be equally as bad. As a consequence, the new emerging model for a functional role of α-Syn may need to consider α-Syn levels in a more dynamic perspective; it appears that there may only be a limited physiological range in which α-Syn levels are beneficial to neuronal function. The collected data so far indicate that excursions to either side are associated with neurological problems.

It will be very intriguing to see what future studies reveal about this possible bimodality of low or high α-Syn levels along a wider spectrum of neurological disorders. If this bimodality is confirmed in other diseases, targeting the iron system could become a viable method in the clinic to identify and control altered levels of α-Syn, based on a patient’s blood serum data. This then would fit into the emerging drive to provide a more personalized and precision-guided treatment approach in the clinic.
